# Depth-selective method for time-domain diffuse reflectance measurements: validation study of the dual subtraction technique

**DOI:** 10.1364/BOE.497671

**Published:** 2023-11-10

**Authors:** Elham Fazliazar, Aleh Sudakou, Piotr Sawosz, Anna Gerega, Michal Kacprzak, Adam Liebert

**Affiliations:** Nalecz Institute of Biocybernetics and Biomedical Engineering, Polish Academy of Sciences, Warsaw, Poland

## Abstract

Research on the spatial distribution of sensitivity of time-domain near infrared diffuse reflectance measurement is reported in this paper. The main objective of the investigation is to validate theoretically calculated sensitivity profiles for a measurement geometry with two detectors and two sources in which sensitivity profiles of statistical moments of distributions of time of flight of photons (DTOFs) are spatially restricted to a region underneath the detectors. For this dual subtraction method, smaller sensitivities to changes appearing in the superficial layer of the medium were observed compared to the single distance and single subtraction methods. Experimental validation of this approach is based on evaluation of changes in the statistical moments of DTOFs measured on a liquid phantom with local absorption perturbations. The spatial distributions of sensitivities, depth-related sensitivity and depth selectivities were obtained from the dual subtraction method and compared with those from single distance and single subtraction approaches. Also, the contrast to noise ratio (CNR) was calculated for the dual subtraction technique and combined with depth selectivity in order to assess the overall performance (product of CNR and depth selectivity) of the method. Spatial sensitivity profiles from phantom experiments are in a good agreement with the results of theoretical studies and feature more locally restricted sensitivity volume with the point of maximal sensitivity located deeper. The highest value of overall performance was obtained experimentally for the second statistical moment in the dual subtraction method (∼10.8) surpassing that of the single distance method (∼8.7). This confirms the advantage of dual subtraction measurement geometries in the suppression of optical signals originated in the superficial layer of the medium.

## 1. Introduction

Near infrared spectroscopy (NIRS) is a non-invasive monitoring technique broadly used in the fields of brain function assessment ranging from investigation of cognitive processes [1] to clinical applications [2,3]. Yet, unique potentials of this technique are not fully explored due to a number of disadvantages. The most important limitation is contamination of measured signals by the components related to the hemodynamic changes appearing in the extracerebral tissue [4–7]. In classical approach –continuous wave NIRS (cw-NIRS), a continuous light source is located on the surface of a head and light propagates through the tissue to the detection spot located a few centimeters away from the source. By monitoring the changes in the intensity of the back-scattered light at multiple wavelengths, it is possible to derive changes in concentration of oxy- and deoxy-hemoglobin appearing in the brain cortex [8]. However, task-evoked and systemic hemodynamic changes, mostly assigned to fluctuations in heart rate, blood pressure, breathing rate, autonomic nervous system (ANS) activity and concentration of CO_2_ in the blood [7] appearing in the superficial tissue layers (scalp and skull) lead to the light absorption changes [4,9] and in consequence to changes in the detected light intensity. These contaminating signals can decrease the accuracy of assessment of hemodynamic changes in the brain cortex using NIRS technique [10,11]. In frequency-domain (fd-NIRS) measurement, the intensity of light emitted into the tissue is modulated, and changes in the phase are monitored in addition to the changes in intensity. The influence of extracerebral tissue layers can be suppressed using fd approach [12,13]. Time-domain method (td-NIRS) is the most informative NIRS technique allowing to distinguish signals originating from the brain cortex [6]. It was shown both theoretically and in experiments carried out on phantoms and in in-vivo validation studies that td-NIRS improves discrimination between superficial and cerebral absorption changes [13,14]. In the td-NIRS approach, ultra-short laser pulses are emitted into the tissue. For individual photons the time spent travelling through tissue to the detector is recorded which allows to build a distribution of time of flight of photons (DTOF). Assuming that the late coming photons are more likely to have penetrated the deeper layers of the tissue (including the brain cortex), different methods of DTOF analysis were proposed offering an effective discrimination of the signal from the deeper layer of the tissue [14–17]. However, the fluctuations in the number of late photons are still to some degree influenced by hemodynamic fluctuations appearing in extracerebral tissue [16].

The multi−distance detection is a frequently used technical approach to address the superficial contamination in cw-NIRS [18], fd-NIRS [19] and td-NIRS [20,21] to reduce the influence of changes in the extracerebral tissue layer. Focusing on the td-NIRS, in this approach, the optical signals measured at two source–detector distances are subtracted [22]. However, the assumption behind the subtraction method is that changes in optical signals measured by NIRS are homogeneously distributed throughout the superficial layer. It has been shown that this assumption is not realistic [23,24] and the inhomogeneous changes throughout the scalp become more problematic as the distance between far and near detectors increases. Also, superficial inhomogeneities like moles, non-uniform distribution of hair, hematoma, smears of blood and etc. [25] may make the results of NIRS method inaccurate or not reproducible [26,27]. It is suggested that such skin imperfections should be avoided when positioning the optodes which influences the flexibility of optode positioning. Moreover, inhomogeneities located deeper such as larger blood vessels and gyri, cannot be seen by eye which makes it challenging to avoid them when positioning the optodes on the surface of the tissue [27]. The depth sensitivity was shown to be significantly improved by single subtraction method [20]. However, further improvement in the suppression of signals related to both homogeneous and inhomogeneous absorption changes appearing in the superficial compartments can improve the potential usefulness of the NIRS technique.

Recently, a novel measurement geometry that we will call dual subtraction (DS) was proposed in which two detectors separated by a fraction of centimeter are sandwiched between two sources [28–30]. Theoretical basis of this approach was introduced by Sawosz et al. [28]. Using the solution of diffusion equation, different features of spatial distribution of sensitivity for this method were presented. The proposed approach is based on td-NIRS measurements and estimation of statistical moments of DTOFs: the total number of photons *N*_tot_, the mean time of flight of photons, and the variance *V* of the DTOF [31]. In this approach the single subtraction (SS) of corresponding statistical moments is calculated for each source and both detectors as reported by Milej et al. [20]. Additionally, results of these subtractions obtained for both sources are averaged. Analysis of spatial profiles of sensitivities to absorption changes for this measurement geometry showed that for mean time of flight of photons and variance of the DTOF, the maximal sensitivities are located deeper than in single distance (SD) td-NIRS and the sensitivity volumes are more confined [28].

In a series of studies, this method based on two sources and two detectors was validated in fd-NIRS, showing that maximal sensitivity can be obtained for absorption changes appearing at a depth of about 5 mm for light intensity and much deeper (about 11 mm) for phase (directly related to changes in mean time of flight). Theoretical analysis for this approach confirms a substantial progress compared to the classical single distance method from which the maximal sensitivity is obtained at a depth smaller than 2 mm for the intensity and smaller than 5 mm for the phase measurement [30]. Also, feasibility of the method for different configurations of sources and detectors was analyzed [29] and more flexibility of optode positioning for imaging purposes was gained. Optimal arrangements allowing to gain adequate signal to noise ratio (SNR) and avoid detector saturation were analyzed [29]. Furthermore, the method was applied to design a CW instrument for robust and calibration free measurements of absorbance spectra [32,33].

Theoretical methods to model light propagation in tissue, either the stochastic methods such as Monte Carlo or deterministic methods such as solutions of diffusion equation, have paved the way for rigorous and reliable prediction of results of new source-detector configurations as well as various methods of data analysis [34–36]. However, these theoretical approaches are limited when modelling more complex realistic geometries especially in the volumes located close to the source or detector [37]. The theoretical predictions can be validated in experiments on physical phantoms in which technical limitations of the proposed methods can be evaluated. The spatial sensitivity of an optical detection scenario can be mapped by introducing local absorption perturbations inside an optically turbid medium [38,39]. Assessment of the changes in light intensity [40] or alterations in the shape of DTOF [39] allow to estimate the spatial profile of the sensitivity of moments of DTOFs to the absorption perturbations.

In this study we have applied local absorption perturbation strategy in order to examine the practical usefulness of the dual subtraction geometry. In particular, we aim to measure changes in statistical moments of DTOFs that result from a small absorbing inclusion, which is moved along all positions in three dimensions. This experiment allowed us to compare local sensitivity properties of the dual subtraction technique with the single-distance and single subtraction measurements. We will show the results of the analysis of the sensitivity together with depth selectivity of statistical moments as defined in [15]. Finally, in order to assess the influence of noise on measurands, we will evaluate the performance of dual subtraction method by combined analysis of depth selectivity and *CNR*, similar as was done in [41] for a quantitative comparison of performance of different measurands.

## 2. Method

### 2.1 Experimental setup

**Fig. 1.**
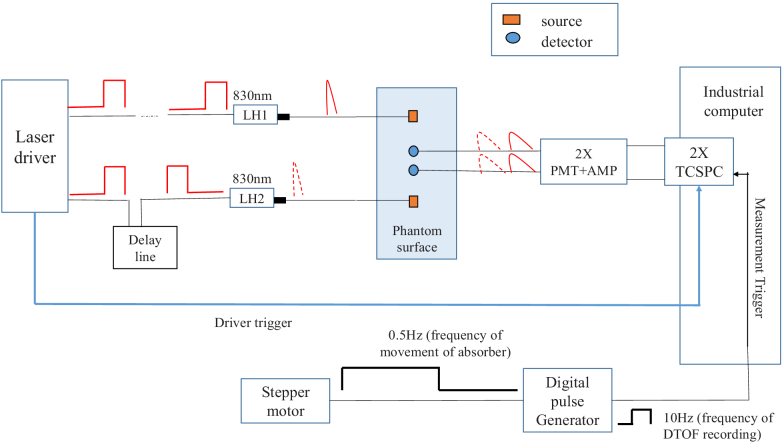
Schematic of the experiment setup consisting of a time-resolved NIRS system with semiconductor laser heads LH1 and LH2, photomultiplier tubes PMT for light detection, preamplifiers AMP and time-correlated single photon counting TCSPC cards for recording of DTOFs

The developed time-domain NIRS system for simultaneous data acquisition from four pairs of source-detectors is shown in Fig. 1. The same system, but with slightly different configuration, was used in previous studies [42]. The setup consists of two semiconductor diode lasers operating at 830 nm (PicoQuant, Germany) controlled using a laser driver (Sepia II PDL 828, PicoQuant, Germany). Light pulses from both laser diodes were generated with the repetition rate of 64 MHz. The light pulse train from the second laser diode was shifted in time by a delay line (Kentech Instrument Ltd, England). This temporal shift allowed us to deliver light pulses sequentially to two locations on the surface of the phantom within a single laser repetition period. Two independent PCI boards with time-correlated single photons counting electronics (SPC-130, Becker & Hickl, Germany) were used for simultaneous acquisition of four DTOFs related to four source-detectors pairs used in this technical approach. Recording of DTOFs during the experiments was triggered by a 10-Hz digital pulse generator (NI-DAQ USB-6210, National Instruments, Austin, Texas). SPC-130 boards and NI-DAQ 6210 were controlled by Becker&Hickl TCSPC software package (version 9.74). The laser pulses were delivered to the phantom by 2 m long bifurcated fiber bundles (BFY400HS02, Thorlabs, Sweden). The measurement system was designed with two bifurcated fibers on the laser side, in order to provide measurements at two wavelengths, as the setup was intended for future in-vivo experiments with estimation of changes in hemoglobin concentrations [20]. However, for the phantom measurements in this study, only one wavelength was utilized, and only one branch of each bifurcated fiber was used. The fiber bundles, which deliver the diffusely reflected light to the detectors, were 3 m long and 3 mm in diameter (Ceram Optec, Germany) with numerical aperture of 0.22. For the single photon detection, we used two high-speed hybrid photodetectors (HPM-100-50 Becker&Hickl, Germany). The instrumental response function (IRF) of the setup was measured by positioning source fibres in front of the detection fibres; the optodes were fixed on opposite walls of a black box with a piece of paper placed in front of the bundles to fill out its numerical aperture [43]. The IRFs measured for all four pairs of source-detector were from 595 ps to 717 ps in full width at half maximum. The measurements were not started earlier than a warm-up time (90 mins) after switching on all the subunits of the system to minimize the instability of laser output.

### 2.2 Phantom experiments

**Fig. 2.**
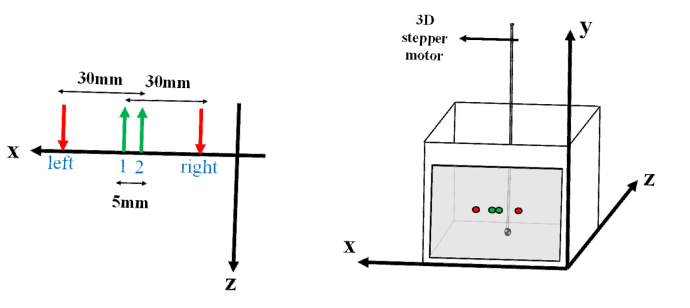
Illustration of the experimental setup: two sources (red arrows with labels “right” and “left”) and two detectors (green arrows with labels 1 and 2) were located on one wall of the fish tank. A black cylinder with a diameter of 3 mm and a height of 5 m was immersed into the phantom by a thin pipe (0.15 mm thickness) which was fixed on a 3D stepper motor.

To prepare the tissue-mimicking phantom, a fish tank (20cm × 20cm × 10 cm) was filled with a solution of milk (3.2% fat) and water, with black ink (KOH-I-NOOR, Czech Rep.) added to obtain optical properties close to those of a living tissue. In order to assess influence of scattering on the DS concept, liquid phantoms with absorption coefficient *µ*_a_≈0.1 cm^-1^ and three different values of reduced scattering coefficients *µ*_s_’≈5, 10 and 20 cm^-1^ were prepared. All the fibers tips (two source fibers and two detecting fibers) were fixed in a 3D-printed optode holder. One wall of the fish tank was constructed of a transparent 50 µm thin Mylar film (DuPont Teijin Films) on which the optode holder was located (*Z *= 0 of Fig. 2). To prevent the Mylar film from changing shape due to the pressure of the liquid phantom, we tied fixers on the outside of the fish tank to hold the film in place. Semi-infinite and homogeneous geometry of measurements were created for all source-detector pairs. The interoptode distances for two pairs of source-detectors were 3 cm and for the other two pairs 2.5 cm (as shown in Fig. 2). The local absorption change in the phantom was introduced using a cylindrical black plastic with a diameter of 3 mm and a height of 5 mm, which needed to be highly absorbing and not to have scattering surface [39]. The absorber was immersed into the liquid by a thin plastic pipe (0.15 mm thickness of walls, 1.5 mm outer diameter and the liquid could flow inside the pipe) as shown in Fig. 2 and moved in the medium using a 3D stepper motor (DMX-J-SA-17, Arcus-technology, USA). The effect of absorption caused by the pipe was measured without the black plastic absorber and it was negligible (sum of contrast caused by the pipe is on average 5% of contrast caused by the black plastic). The stepper motor made a single step every 2 seconds and swept a cuboid of dimensions 75 mm (along X axis) × 35 mm (along Z axis) × 20 mm (along Y axis) (Fig. 2). The upper surface of this cube was located parallel to the XZ plane and intersects a line containing the optodes. The steps along horizontal axes X and Z were 2mm-long, and along the vertical Y axis - 1mm-long. The accuracy and repeatability of steps of the absorber movement were 10 microns and ±5 microns respectively. The stepper motor was triggered by a digital pulse train generated at a frequency of 0.5 Hz. This digital pulse train was generated by the National Instrument Card used in the time-domain system and, thus, was synchronized with data acquisition triggering of this system. Twenty DTOFs from each pair of source-detector were recorded for each single positioning of the absorber. The frequency of data acquisition triggering was 10 Hz (data collection time was 0.09 s to assure enough time for data saving). The total count rate was around 3.5 million on average for the sum of two DTOFs (recorded by each detector from two sources) per second.

### 2.3 Data analyses

The measurement system presented in Fig. 1 allowed to record DTOFs for four source-detector pairs according to the optode configuration shown in Fig. 2. The optical properties of prepared homogeneous phantom were estimated prior to immersing the absorber using the statistical moments of measured DTOFs according to the equations presented in [43]. The reduced scattering coefficient *µ*_s_’ was ≈10 cm^-1^ (10.13 cm^-1^) and absorption coefficient *µ*_a_ was ≈0.1 cm^-1^ (0.098 cm^-1^). Next, the absorber was immersed and started the movement. From each group of 20 DTOFs recorded continuously and sequentially for each position of the absorber, 10 first DTOFs which were recorded during the movement period of the stepper motor were excluded from the data analysis. The average of the next 10 DTOFs was calculated for each position of the absorber for further analysis. In the calculation of the statistical moments, both sides of DTOFs were cut at a point where the number of counts dropped below 1% of its maximum value. Three statistical moments (*N*_tot_, <*t *> and *V*) of each sampled DTOF were calculated for each position of the absorber. Elements of a 3D matrix were filled with each of the three moments, in a sequence according to the movement pattern of thstepper motor (three 3D matrices were created for three statistical moments; *N*_tot_, <*t *> and *V*). The sums of 3D matrices over a selected dimension allowed to reconstruct images of spatial distributions of the different statistical moments on different coordinate planes. Average of statistical moments of DTOFs when the absorber was located on the outermost side of the swept volume was calculated as the statistical moments of the homogeneous phantom. Then, according to the formulas 1-3, math operations were conducted on each element of the obtained 3D matrix to calculate the distributions of changes in the statistical moments caused by the small absorption perturbation Δ*µ*_a_.

(1) $$\Delta A=-\log\frac{N_{\text{tot},\text{i}}}{N_{\text{tot},0}}$$

(2) $$\Delta <t>\;=\;<t{>}_{0}-<t{>}_{i}$$

(3) $$\Delta V=V_{0}-V_{\text{i}}$$

In these equations, *N*_tot,0_, <*t *> _0_ and *V*_0_ are statistical moments of the homogeneous phantom and *N*_tot,i_, <*t *> _i_ and *V*_i_ are statistical moments of the phantom when the absorber was in a defined small volume indexed by *i*. Afterwards, spatial distributions of changes in statistical moments obtained from the dual subtraction method were generated. According to the dual subtraction concept [28], changes in statistical moments for each source (red arrows on the right and left sides in Fig. 2) and two detectors were subtracted and then the results of these subtractions for two sources were averaged. Formulas 4-6 are utilized to derive a moment *M* changes in the dual subtraction method:

(4) $$\Delta M_{\text{ss\_left}}=\Delta M_{\text{left\_2}}-\Delta M_{\text{left\_1}}$$

(5) $$\Delta M_{\text{ss\_right}}=\Delta M_{\text{right\_1}}-\Delta M_{\text{right\_2}}$$

(6) $$\Delta M_{DS}=\frac{1}{2}(\Delta M_{\text{ss\_left}}+\Delta M_{\text{ss\_right}})$$

The index *left* or *right* refers to the left or right source and the index *1* or *2* refers to the detector *1* or *2* in Fig. 2. Δ*M*_SS_left_ and Δ*M*_SS_right_ denote Δ*M* obtained from single subtraction method which is a result of subtraction of Δ*M* obtained by small source-detector separation (2.5 cm) from Δ*M* obtained by large source-detector separation (3 cm). Δ*M*_DS_ denotes the final changes of the statistical moment *M* obtained from dual subtraction method.

During the measurements, an intensity cross-talk between source-detector pairs was observed. The cross-talk was between two source-detector pairs with a common detector that were separated by the delayed excitation of laser sources. In such laser multiplexing, the counting loss due to dead-time in the signal resulting from one laser source affects the signal resulting from the other laser [44]. A correction of the number of photons using the formula for dead-time counting loss was applied [44] which made the observed cross-talk negligible.

In the next step, depth-related sensitivity was studied which is sensitivity to a homogeneous absorption change throughout a defined layer of the medium. It was estimated by summing up the elements of matrices (changes in the statistical moments of the DTOFs) along the Y axis and then once again along the X axis. Thus, the 3D matrix changed into a column vector along the Z axis (depth in the phantom, see Fig. 2). Furthermore, depth selectivity of the changes in the statistical moments was calculated. As defined in Ref. [15], depth selectivity (*S*) of a measurand *M* was calculated as the ratio of total sensitivity of the measurand *M* to absorption changes within the deeper and the upper layers of same thickness.

(7) $$S_{\text{M}}=\frac{\sum_{i=lower}SF_{\text{M, i}}}{\sum_{i=upper}SF_{\text{M, i}}}$$

In this equation *SF* denotes sensitivity factor which is equal to changes in the statistical moments of DTOF divided by the change of absorption in the defined small volume indexed by *i* caused by the absorber (black ball) [31].

(8) $$SF_{\text{M,i}}=\frac{\Delta M_{\text{i}}}{\Delta \mu_{\text{a, ball}}}$$

Since the change in absorption caused by the black plastic ball (Δ*µ*_a,ball_) is the same for the all voxels, Δ*µ*_a,ball_ is cancelled out from the numerator and denominator of Eq. (7) and it is not necessary to measure Δ*µ*_a,ball._ The thickness of upper and lower layers that we chose is ≈5 mm as in [15]. To calculate *S*_M_ as a function of the depth of lower layer, sum of sensitivity within a layer of ≈5 mm starting from a depth of *Z* (*Z* moves from 5 mm to 30 mm) was divided to the sum of sensitivity within a layer of ≈5 mm starting from the surface (*Z*≈0).

Finally, the overall performance of the method was evaluated using a parameter which is the product of depth selectivity and *CNR* [41]. This product allows for quantitative comparison of the influence of both superficial contamination and uncertainty among different data analysis methods.

(9) $$S_{\text{M}}\times CNR_{\text{M}}=\frac{{\left(\sum_{i=lower}SF_{\text{M, i}}\right)}^{2}}{\sigma \;(M_{0})\times \sum_{i=upper}SF_{\text{M, i}}}$$

It is important to note that the sum of sensitivity caused by the black plastic ball within a layer cannot be equivalent to layered sensitivity due to a significant absorption change that goes beyond the linear perturbation theory. However, this summation can still be utilized for relative comparisons of different data types and source-detector arrangements. In Eq. (9), $\sigma \;(M_{0})$ denotes standard deviation of the measurand due to photon noise in the homogeneous medium. Theoretically, these standard deviations for the three statistical moments and for the single distance method have been derived as follows [45].

(10) $$\sigma^{\;2}(N)=\sum N_{\text{i}}=N$$

(11) $$\sigma^{2}(m_{1})=\;\frac{V}{N}$$

(12) $$\sigma^{2}(V)=\frac{m_{4}-\;V^{2}}{N}$$

All the statistical moments presented in Eqs. (10)–(12) are valid for homogeneous medium and m_i_ is the *i*th centralized statistical moment of the DTOF. According to the error propagation principle, for the single subtraction method with two pairs of source-detector, for any statistical moment, assuming that *σ*_SD,far_ = *σ*_left,2_ = *σ*_right,1_ and *σ*_SD,near_ = *σ*_left,1_ = *σ*_right,2_ (see Fig. 2):

(13) $${\sigma^{2}}_{\text{SD}}={\sigma^{2}}_{\text{SD,far}}+{\sigma^{2}}_{\text{SD,near}}$$

Since dual subtraction is an average of two single subtraction methods,

(14) $${\sigma^{2}}_{\text{DS}}=\frac{1}{2}({\sigma^{2}}_{\text{SD,far}}+{\sigma^{2}}_{\text{SD,near}})$$

Here, the indices SS, DS and SD referred to the single subtraction, dual subtraction and single distance geometries, respectively. The indices *far* and *near* referred to the channel with larger and smaller source-detector separations, respectively. Therefore, the theoretical photon noise for the dual subtraction method is $\frac{\sqrt{2}}{2}$ of the single subtraction method and slightly smaller than single distance method since noise is correlated with source-detector distance (*σ*^2^_SD,near_ < *σ*^2^_SD,far_).

## 3. Results and discussion

**Fig. 3.**
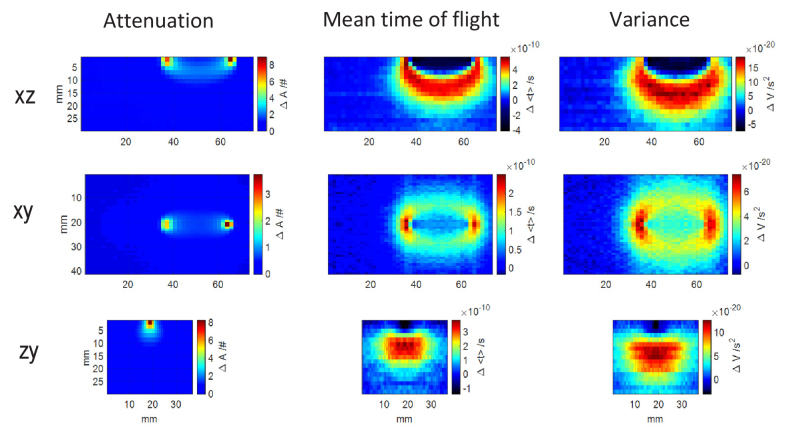
Spatial distributions of changes in the statistical moments of DTOF (number of photons, mean time of flight and variance) obtained for source-detector separation of 3 cm along X axis presented on three coordinate planes for a phantom with *µ*_a_≈0.1 cm^-1^ and *µ*_s_’≈5 cm^-1^.

The spatial distributions of changes in three statistical moments (Δ*A*, Δ<*t *> and Δ*V*) in three coordinate planes (XZ, YZ and XZ) for a source-detector pair of 3 cm separation are presented in Fig. 3 for a phantom with *µ*_a_≈0.1** **cm^-1^ and *µ*_s_’≈5** **cm^-1^. All three statistical moments form the so-called banana shaped sensitivity profile in XZ plane and sensitivity throughout this banana is not uniformly distributed. As expected, sensitivity is shifted towards deeper compartment and becomes more uniform within the banana as the order of statistical moment increases [15,41]. Δ*A* is always positive and shows its highest values close to the surface of the medium, especially close to the positions of source and detector. Therefore, it is more sensitive to the homogeneous and regional changes appearing in the superficial compartment of the medium [46]. First and second statistical moments have negative values in regions located between optodes close to the surface of medium and reveal maximal sensitivity in deeper compartments of the medium. Therefore, as compared to Δ*A*, Δ<*t *> and Δ*V* are more sensitive to changes in absorption appearing in the deeper layer of medium [46]. This is consistent with results from the in-vivo studies that compared to number of photons, mean time of flight and variance are more sensitive to changes in absorption appearing in the brain cortex and less sensitive to changes appearing in the superficial layer of head tissue [46].

**Fig. 4.**
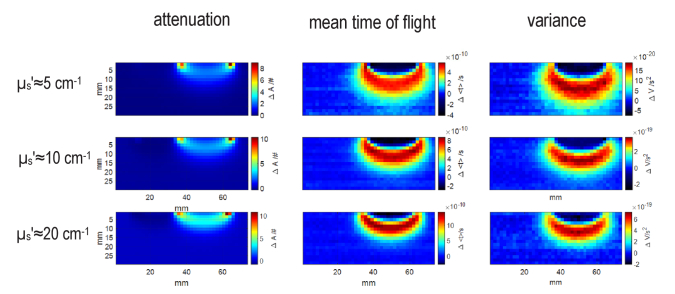
Spatial distributions of changes in the statistical moments of DTOF obtained for source-detector separation of 3 cm for µ_a_≈0.1 cm^-1^ and three different µ_s_’≈5, 10 and 20 cm^-1^.

Reduced scattering coefficient is a parameter which significantly influences the diffuse reflectance measurement [47]. This quantity is wavelength-dependent and should be considered when choosing the wavelength to obtain improved image resolution [48]. The spatial distribution of changes in three statistical moments for a phantom with absorption coefficient *µ*_a_≈0.1 cm^-1^ and three different reduced scattering coefficients *µ*_s_’≈5, 10 and 20 cm^-1^ are presented in Fig. 4 for source detector separation of 3 cm. As can be seen, the volume of high sensitivity becomes smaller and is located closer to the surface as the reduced scattering coefficient increases. For the phantoms with equal absorption coefficient, the penetration depth depends on the attenuation caused by scattering. For higher scattering coefficient, the penetration in all directions is reduced which results in more concentrated sensitivity profile and maximal sensitivity located closer to the surface of medium.

It is observed in Fig. 4 that the thickness of layer with negative sensitivity in mean time of flight and variance for SD method is almost independent from the value of reduced scattering coefficient. For all statistical moments, contrast is raised with an increase in reduced scattering coefficient. However, it was shown that, the higher contrast does not compensate for higher noise and shallower maximal sensitivity in the highly scattering medium [48]. Therefore, in diffuse reflectance measurement, absorption changes appearing in the deeper compartment are more detectable in the lower scattering media.

**Fig. 5.**
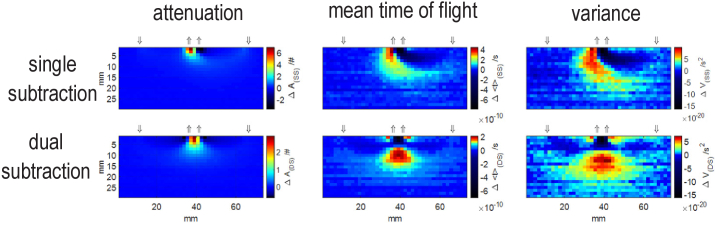
Spatial distributions of changes in statistical moments of DTOF for a phantom with *µ*_a_≈0.1 cm^-1^ and *µ*_s_’≈5 cm^-1^. Top row: single subtraction for the source located on the right side and pair of detectors. Bottom row: sum of subtractions obtained for two sources located on the right and left sides of the pair of detectors.

The top row in Fig. 5 presents results of single subtraction analysis with one source and two detectors. Subtraction method can be implemented equivalently with either two sources and one detector or two detectors and one source, provided that the size of the illumination and detection spots are equal and the NA on both sites of the system are the same. The single subtraction (SS) method has been shown to shift the maximal sensitivity to deeper layers in td-NIRS [20] and fd-NIRS [49]. It also reduces the effect of motion artefacts or dependence on the inappropriate tissue-optode contact [50–52]. This advantage can especially be crucial in long term or motor task measurements in which optodes are more exposed to be moved.

As can be seen in Fig. 5, the maximum positive and negative sensitivities in the single subtraction (SS) method are located close to the surface. Considering the changes of absorption that are distributed uniformly in the superficial compartment, the positive sensitivity in the superficial layer is cancelled out by the negative sensitivity which causes the single subtraction method be negligibly sensitive to the superficial layer. However, these strong local sensitivities still leave the method highly affected by local inhomogeneity in the superficial layer. For the single subtraction method, a broad sensitivity profile is observed whereas the dual subtraction method can improve the spatial resolution of measurement (Fig. 5). The dual subtraction method inherits all the above-mentioned benefits of the single subtraction method. Furthermore, the following advantages of dual subtraction method reported in the theoretical work [28] are observed in Fig. 5: (1) more localized positive sensitivity in all three statistical moments which results in improved spatial resolution of the measurement compared to both single distance (SD) and single subtraction (SS) methods, (2) the negative superficial sensitivities in Δ<*t *> and Δ*V* as shown in the bottom row of Fig. 5 are more localized, (3) the problem of strong local sensitivities in the superficial layer is modified to a good extent and the asymmetric sensitivity profile in the single subtraction method is solved through the symmetric setup in the dual subtraction method [29].

**Fig. 6.**
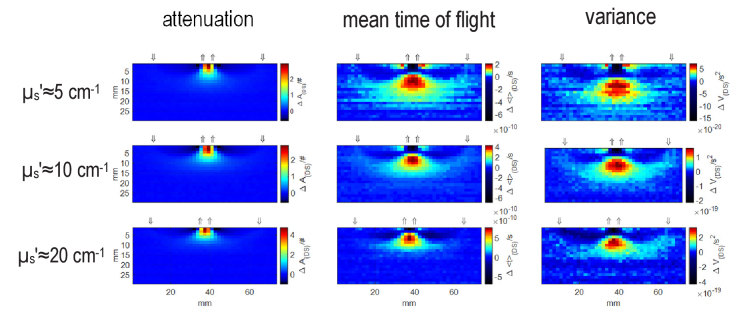
Spatial distributions of the dual subtractions method for three statistical moments of DTOFs obtained for a phantom with *µ*_a_≈0.1 cm^-1^ and three different values of reduced scattering coefficients *µ*_s_’≈5,10 and 20 cm^-1^.

Depth resolution in td-NIRS depends on the reduced scattering coefficient [31]. In order to assess influence of scattering on the DS concept, the analysis was repeated for the liquid phantoms with *µ*_a_≈0.1 cm^-1^ and three different values of reduced scattering coefficients *µ*_s_’≈5, 10 and 20 cm^-1^. The spatial distributions of changes in three statistical moments for three different scattering coefficients resulted from dual subtraction method are shown in Fig. 6. The distributions of changes for the dual subtraction method presented in Fig. 6 agree with the distributions for single distance method shown in Fig. 3 in terms of relation between maximal depth sensitivity and the order of statistical moments. The highest sensitivity compartment is shifted deeper as the order of statistical moment increases for all values of the reduced scattering coefficient. Moreover, same to the single distance method (shown in Fig. 4), the contrast increases with the increase in scattering coefficient. The more localized sensitivity in the profile observed for *µ*_s_’≈20 cm^-1^ agrees with the more concentrated sensitivity for a highly scattering medium in the single distance method (Fig. 4).

**Fig. 7.**
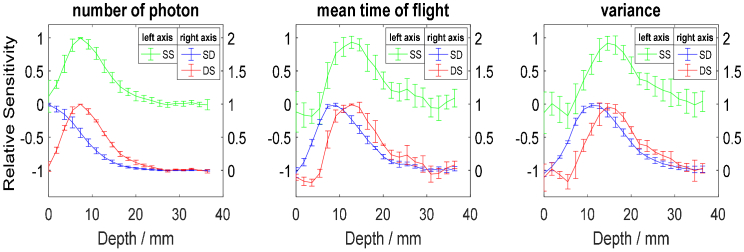
Normalized depth-dependent sensitivities of statistical moments to absorption change appearing a layer of 2 mm thickness for a single-distance method with source-detector separation of 3 cm (*µ*_a_≈0.1 cm^-1^ and *µ*_s_’≈10 cm^-1^) and the single and dual subtraction methods with large source-detector separation of 3 cm and small source-detector separation of 2.5 cm.

Starting here, we have shifted our focus to evaluating layer-based relative sensitivity rather than voxel-based sensitivity. We extended the sensitivity analysis of statistical moments of DTOF in dual subtraction method [28] here by studying the relative sensitivity as a function of depth in the medium and depth selectivity. The experiment was repeated four times for a phantom with *µ*_a_≈0.1 cm^-1^ and *µ*_s_’≈10 cm^-1^. For each experiment spatial distributions of changes in statistical moments were generated for two single distance with 3 cm source-detector separation, two single subtractions and one dual subtraction geometries. Sums of sensitivities within the layers of 2 mm thickness parallel to XY plane (Fig. 2) for statistical moments were calculated in order to assess the depth-related sensitivity assuming a uniform absorption change in every layer. Then these depth-related sensitivities were normalized by dividing the sensitivity of each layer by the layer with maximal sensitivity. Mean of the normalized depth-related sensitivities from four experiments and corresponding standard deviations are shown in Fig. 7 resulted from eight single-distance (SD), eight single subtraction (SS) and four dual subtraction (DS) curves. Even though, there are regions with negative sensitivity in the spatial sensitivity profiles of first and second statistical moments of single-distance measurement (as shown in Fig. 4), sensitivities summed within layers are always positive. Whereas in dual subtraction method, depth-dependent sensitivity is slightly negative in the superficial layer for variance and mean time of flight (Fig. 7). The primary purpose of DS method is to suppress the sensitivity in superficial layer rather than differentiating it from the sensitivity of deep layer. For such method, the ideal case is zero sensitivity (or non-sensitivity) to changes in absorption appearing in the superficial layer. The negative sensitivity in superficial layer still can be as problematic as positive sensitivity and lead to wrong estimation of the absorption changes originated in the deeper compartment of the medium in diffuse reflectance measurement. However, the amplitude of sensitivity to changes appearing in the superficial compartment in dual subtraction is small (almost non-sensitive) compared to single distance method (Fig. 7) which shows that the dual subtraction method can suppress changes originated in superficial layer more effectively.

Spatial distribution of localized sensitivity has been simulated by other groups for different arrays of optodes that fulfill the requirements for the dual subtraction method [28,29]. These different optode configurations result in different spatial sensitivity profiles. However, regardless of the arrangement of optodes, the depth related sensitivity for single subtraction approach coincides with dual subtraction method which means that the maximal sensitivity of both methods is located at the same depth. The reason of this coincidence is that for a uniform absorption change the signal from dual subtraction method is equal to the average of two single subtraction methods and averaging signals from the same method does not change the depth of maximal sensitivity. Despite the similarity of the sensitivity profiles of single and dual subtraction methods (see Fig. 7), it can be seen that standard deviation in dual subtraction method is smaller than the single subtraction method which is because averaging signals in general decreases the noise. In all three considered methods (SD, SS and DS) the compartment of maximal sensitivity moves deeper as the order of statistical moment increases. The more prominent feature which can be observed in Fig. 7 is that the maximum of sensitivity appears in deeper layer in dual subtraction method compared to single distance method. The improvement in the zeroth and first statistical moments here agrees with results of analysis of intensity and phase (as equivalent measurand of mean time of flight of photons) acquired in frequency domain measurements [30]. The second centralized statistical moment features the maximum of sensitivity in the compartment located deeper in the medium as compared to the zeroth and first statistical moments for single distance method. Correspondingly, this feature is observed in the dual subtraction method. It can be seen in Fig. 7 that this measurement geometry with two sources and two detectors is also beneficial for the analysis of changes in light intensity. This can be of high importance since most of the commercially available NIRS systems are continuous-wave and only provide information on changes in the light intensity. Noticeably larger standard deviation in dual subtraction method compared to single distance method and smaller contrast in dual subtraction method (due to the subtraction of signals) can be pointed out as the limitation of this method since it leads to smaller *CNR* compared to the single distance method.

**Fig. 8.**
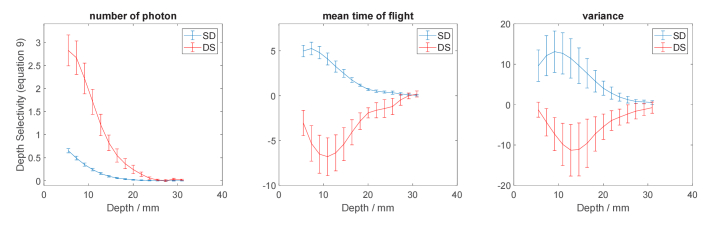
Depth selectivity of statistical moments for single-distance method with source-detector separation of 3 cm and dual subtraction method (*µ*_a_≈0.1 cm^-1^ and *µ*_s_’=10 cm^-1^) with large source detector separation of 3 cm and small source-detector separation of 2.5 cm.

In next step, depth selectivity (Eq. (9)) of statistical moments for the single-distance measurement and the dual subtraction method were calculated for the same four repeated experiments. Average of the resulted depth selectivity curves with corresponding standard deviations are presented in Fig. 8. We have chosen not to present the depth selectivity results for the single subtraction method in Fig. 8 due to significant noise interference. Theoretically, the depth selectivity curves for single and dual subtraction methods coincide since dual subtraction is an average of two single subtraction methods.

For both single distance and dual subtraction methods, standard deviation in Fig. 8 becomes larger as the order of statistical moments increases. For the single distance method depth selectivity is always positive for all three statistical moments even though there are negative sensitivity points for mean time of flight and variance as shown in Fig. 6. Positive depth selectivity means that changes in absorption appearing in the superficial and deep regions will affect the signal in the same direction. On the contrary, in the dual subtraction method, depth selectivity for mean time of flight and variance is always negative which is due to negative values of sensitivity in the superficial layers (Fig. 7). Thus, the signs of sensitivity are opposite for changes in absorption appearing in the superficial and deep compartments. Larger depth selectivity means increased contribution of deeper layer in the merged signal. Larger depth selectivity in td-NIRS was shown to improve the accuracy in reconstruction of *µ*_a_ [20]. Accordingly, four prominent features observed in Fig. 8 are (1) the magnitude of selectivity becomes greater as the order of statistical moment increases for both single distance and dual subtraction methods; (2) for all three approaches maximal depth selectivity moves towards deeper compartment as the order of statistical moment increases; (3) maximum of selectivity is noted in the compartments located deeper in the dual subtraction method for mean time of flight and variance compared to the single distance method; (4) considerably higher depth selectivity was achieved through dual subtraction method for number of photons compared to single distance method. While td-NIRS can take the advantage of analysis of late time windows [15,49], the only way to obtain improved depth selectivity in cw-NIRS is using distance resolved method [50]. Accordingly, the improvement gained by dual subtraction method on the zeroth statistical moment (number of photons) which is equivalent to light intensity in cw-NIRS is of high importance. The maximal depth selectivity from dual subtraction method for measurement of total number of photons is over twice as large as that of single distance method. This finding supports the results of previous studies [30] in terms of improvement in sensitivity and depth selectivity in case of analysis of light intensity. Therefore, the method may boost the results obtained from continuous wave NIRS (cw-NIRS) approach. The curves of relative depth-dependent sensitivity and depth selectivity for the single distance measurement (presented in Fig. 7 and Fig. 8) were in general agreement with the theoretical findings by Wabnitz et al. [15]. However, slight differences were observed. Other than the well-known sources of discrepancies between simulations and experiments such as refractive index match, different boundary conditions etc. [37,51], we assigned the observed differences to the larger size of absorber used in experiments compared to simulations, the different size of voxels, and noise contribution. We carried out simulations using diffusion equation solution with different size of absorber and found that larger size of absorber while increasing the magnitude of local and depth-related sensitivity, decreases the depth of maximal sensitivity, depth of maximal depth selectivity and the magnitude of depth selectivity. Same effect was reported for magnitude and distribution of layered sensitivity in a theoretical and experimental study [52]. Furthermore, after adding noise to the simulated data, it was observed that due to noise, depth selectivity changes and becomes unrepeatable when thin superficial layer (≈5 mm) is assumed. It should be noted that the discrepancy between theoretical and experimental results may also arise from the experimental use of an infinitely absorbing ball, instead of the linear perturbation adopted in the theoretical work.

**Fig. 9.**
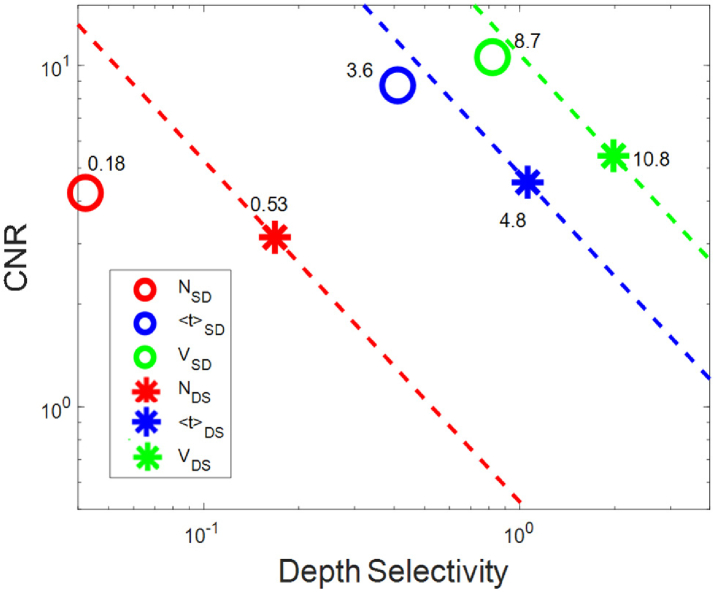
Comparison of the overall performance of single distance and dual subtraction methods. Values of the product of depth selectivity and *CNR* (both unitless parameters) are shown beside each point. The dashed lines show all the points in the graph wi with the same value of the product of *CNR* and depth selectivity. The thickness of the top layer is around 15 mm for the phantom with *µ*_a_≈0.1 cm^-1^ and *µ*_s_’≈10 cm^-1^.

In the final step, the trade-off between *CNR* and depth selectivity for the dual subtraction method was considered for the first time. In single distance method, depth selectivity is improved as the order of statistical moment increases. However, *CNR* decreases [41] which makes the moments higher than 2^nd^ order not commonly used measurands in td- NIRS. In the same way, the smaller contrast in dual subtraction method (due to subtraction of signals from near and far detectors) may impede the practical utility of the dual subtraction method in realistic situation. Despite being beneficial, depth selectivity has been proved not to be a sufficient parameter to evaluate the performance of a method. Both distribution (maximal depth) and magnitude of depth selectivity are independent factors from *CNR*; it is possible that a measurand features a high depth selectivity but is not practically useful due to low *CNR*. It was shown that using the time channels in td-NIRS, ratios of the photon counts in late time windows may offer a higher depth selectivity than statistical moment approach [15]. However, after extending the analyses by including noise this conclusion was not reproduced and it was found that statistical moments are more practically useful than other measurands such as the number of photons in time windows or their ratios [41]. Therefore, other than the depth selectivity, the influence of noise seems essential to be considered when evaluating a method. In the Fig. 9 both depth selectivity and *CNR* resulted from the dual subtraction method are compared with the single distance method for three statistical moments. We assumed ≈15 mm for thickness of the top layer since the maximum of typical range of thickness of the scalp and skull is around this value [53]. As can be seen from Fig. 9, variance features the highest value of the product of depth selectivity and *CNR* in both single distance and dual subtraction methods. This is consistent with the results of the study [41], where it was found that variance has the highest product for single distance method. The dashed lines are obtained by multiplying *CNR* and depth selectivity obtained from the experiments. These lines guide the readers to see the improvement of overall performance (product of *CNR* and depth selectivity) in DS method compared to SD method. All points on each of the dashed lines have equal values of overall performance. As a line locates further from the coordinate origin, it represents a larger value of *CNR* × depth selectivity meaning a better overall performance.

For all the statistical moments, dual subtraction offers higher product of depth selectivity and *CNR* (∼ 0.53 for zeroth statistical moment, ∼ 4.8 for first statistical moment, and ∼ 10.8 for second statistical moment) compared to the single distance method (∼ 0.18 for zeroth statistical moment, ∼ 3.6 for first statistical moment, and ∼ 8.7 for second statistical moment). Therefore, the smaller *CNR* in the dual subtraction method does not overshadow its improved depth selectivity. However, the physiological noise in in-vivo studies is not included in this study [54] and the vulnerability of method to the noise is one of significant limitations. The values shown in Fig. 9 were obtained for specific source-detector separation used in this study and for a homogeneous medium. It should be noted that heterogeneous phantom (with varying optical properties along the depth) can have impact on the depth selectivity and consequently on the overall performance of the DS approach. In our future research, we plan to explore the optimization of the dual subtraction method by examining the depth selectivity, *CNR*, and spatial resolution considering non-homogeneous layered medium. Our aim is to conduct a quantitative comparison among the single distance, single subtraction, and dual subtraction methods using various optode configurations. Furthermore, we will focus on studying the advantages of the dual subtraction method in monitoring and imaging of dynamic changes in human brain oxygenation. The experimental setup developed for this study allows for collecting data from all source-detector pairs simultaneously at sampling rate of 10 Hz which will allow to use it for the *in-vivo* measurements even for monitoring of dynamic changes in brain tissue oxygenation.

## 4. Conclusions

The research carried out was focused on experimental validation of the approach proposed in [28] and further elaborated on its depth selectivity features and CNR limitations. We conducted liquid tissue-mimicking phantom experiments and adopted local absorption perturbation strategy to demonstrate and examine the practical aspects of the method with the analysis of photon noise. Experimental results from this study are in agreement with the predictions attained from the theoretical study [28] in terms of more localized positive sensitivity gained through dual subtraction method which is confined to a region underneath the pair of detectors and more localized negative superficial sensitivity. We conclude that the dual subtraction method can be practically used. However, the influence of noise effect as a typical limitation of the time-domain NIRS systems should be considered in analysis of the robustness of the proposed approach. We compared depth selectivity of the dual subtraction (DS) method with single subtraction (SS) and single distance (SD) methods. As expected from the theoretical considerations, spatial sensitivity distributions for statistical moments of DTOFs acquired experimentally and analyzed using dual subtraction algorithm showed a negative sensitivity for changes in absorption appearing in superficial layers of the medium whereas they have positive and significantly larger sensitivity to changes located in the deeper layer. For all the studied statistical moments of DTOFs, maximum of sensitivity and selectivity are located deeper in dual subtraction method compared to single distance method. Higher absolute value of depth selectivity for zeroth and first statistical moments is achieved through dual subtraction method. This effect may allow to reach increased sensitivity of the NIRS measurements to the hemodynamic changes appearing in the brain cortex. We observed a smaller contrast and smaller *CNR* in dual subtraction method compared to single distance method. However, the superior overall performance is noted compared to single distance method when analysis of combined parameter (product of depth selectivity and *CNR*) is used. Among the all considered data types in this study the second statistical moment gained from dual subtraction method achieved the highest value of overall performance (∼10.8) compared to the single distance method (∼8.7). The strong positive and negative local superficial sensitivities in the single subtraction method can be problematic in the case of non-homogeneous superficial absorption changes. These locally non-homogeneous sensitivities are diminished through the application of the dual subtraction method. Furthermore, the sensitivity profile obtained through the dual subtraction method is more localized compared to the single-distance and single subtraction methods for all studied statistical moments of DTOFs. This can result in improved spatial resolution of NIRS method. Moreover, the dual subtraction method makes use of average of two single subtraction values. Therefore, it will reduce dependence of measured signals on motion artefacts and inappropriate tissue-optode contact. We presented a td-NIRS experimental setup paper that provides simultaneous data collection for all four source-detector pairs required for the dual subtraction configuration. The instrument is applicable for *in-vivo* measurements enabling signals acquisition at sampling rate of 10 Hz.

## Data Availability

Data underlying the results presented in this paper may be obtained from the authors upon reasonable request.
